# Assessment of anti-MDA5 antibody as a diagnostic biomarker in patients with dermatomyositis-associated interstitial lung disease or rapidly progressive interstitial lung disease

**DOI:** 10.18632/oncotarget.19050

**Published:** 2017-07-06

**Authors:** Liubing Li, Qian Wang, Xiaoting Wen, Chenxi Liu, Chanyuan Wu, Funing Yang, Xiaofeng Zeng, Yongzhe Li

**Affiliations:** ^1^ Department of Rheumatology and Clinical Immunology, Peking Union Medical College Hospital, Chinese Academy of Medical Sciences and Peking Union Medical College, Key Laboratory of Rheumatology and Clinical Immunology, Ministry of Education, Beijing, China; ^2^ Department of Medical Laboratory, The First Hospital of Jilin University, Changchun, China

**Keywords:** anti-MDA5, dermatomyositis, ILD, RPILD, diagnosis

## Abstract

Anti-melanoma differentiation-associated protein 5 (MDA5) antibody have been found in dermatomyositis (DM)-associated interstitial lung disease (DM-ILD) and DM-associated rapidly progressive ILD (DM-RPILD). Due to the conflicting results regarding the association between anti-MDA5 antibody and DM-ILD or DM-RPILD and the diagnostic value of this antibody for DM-ILD and DM-RPILD, we performed this meta-analysis. A systematic search was performed to identify studies published to January 14, 2017. Sixteen publications with 491 DM with ILD versus 605 DM without ILD, as well as eighteen publications with 186 DM with RPILD and 790 DM without RPILD were included. The pooled sensitivity, specificity, and area under the curve (AUC) values of anti-MDA5 antibody for DM-ILD were 0.47 (95% CI: 0.37–0.57), 0.96 (95% CI, 0.92–0.97), and 0.90 (95% CI: 0.88–0.93), respectively, with a low sensitivity value. The pooled sensitivity, specificity, and AUC values were 0.83 (95% CI: 0.77–0.88), 0.86 (95% CI: 0.80–0.91), and 0.87 (95% CI: 0.84–0.90) for DM with RPILD versus without RPILD with good sensitivity and specificity values. Trial sequential analysis showed sufficient evidence to support that anti-MDA5 antibody was associated with DM-ILD and DM-RPILD. The statistical power of this study calculated using G*Power version 3.1.9.2 was more than 99% (α = 0.05). Taken together, these findings suggest that anti-MDA5 antibody has a potential useful ability as a noninvasive biomarker in the diagnosis of RPILD in patients with DM.

## INTRODUCTION

Classic dermatomyositis (CDM) and clinically amyopathic dermatomyositis (CADM) are two classifications of dermatomyositis (DM) and are characterized by the involvement of skeletal muscles, skin, and other organs, especially the lungs [1–3]. Interstitial lung disease (ILD) is an intractable and fatal complication of patients with DM, which can result in higher morbidity and mortality than DM patients without ILD deterioration [4–7]. According to the International Consensus Statement of Idiopathic Pulmonary Fibrosis of the American Thoracic Society and the European Respiratory Society [8], rapidly progressive ILD (RPILD) including acute/subacute interstitial pneumonia is a progressive deterioration associated with ILD within 3 months. The survival time was significantly reduced in myositis patients with RPILD [9]. Therefore, the early diagnosis and timely treatment of DM-ILD and DM-RPILD are important.

Serum myositis-specific autoantibodies (MSAs) are useful markers for the diagnosis of DM and are associated with distinct clinical phenotypes [10]. One of the MSAs, anti-MDA5 antibody, has been reported to be associated with DM-associated ILD (DM-ILD) with an unfavorable survival rate (90-day survival rate: 66.7%) [11]. Anti-MDA5 antibody, also known as anti-CADM-140 antibody, recognizes a retinoic acid induced gene I-like receptor involved in innate immunity, the interferon induced with helicase C domain protein 1 (IFIH1) [12, 13]. Studies show that there are correlations of anti-MDA5 antibody with ILD [14, 15] and RPILD [16, 17] in DM patients.

However, there are controversies regarding the association between the presence of anti-MDA5 antibody and the pulmonary involvement phenotypes of DM patients, as well as the diagnostic accuracy of this antibody for DM-ILD and DM-RPILD. For example, Chen et al. reported that anti-MDA5 antibody was associated with DM-associated-RPILD (DM-RPILD) [18], while Fiorentino et al. reported that no significant association was observed between anti-MDA5 antibody and RPILD [19]. Thus, this meta-analysis was performed to analyze published data on the association of anti-MDA5 antibody with DM-ILD and DM-RPILD, as well as the diagnostic value of this antibody for these diseases.

## RESULTS

### Search results and study characteristics

**Figure 1:**
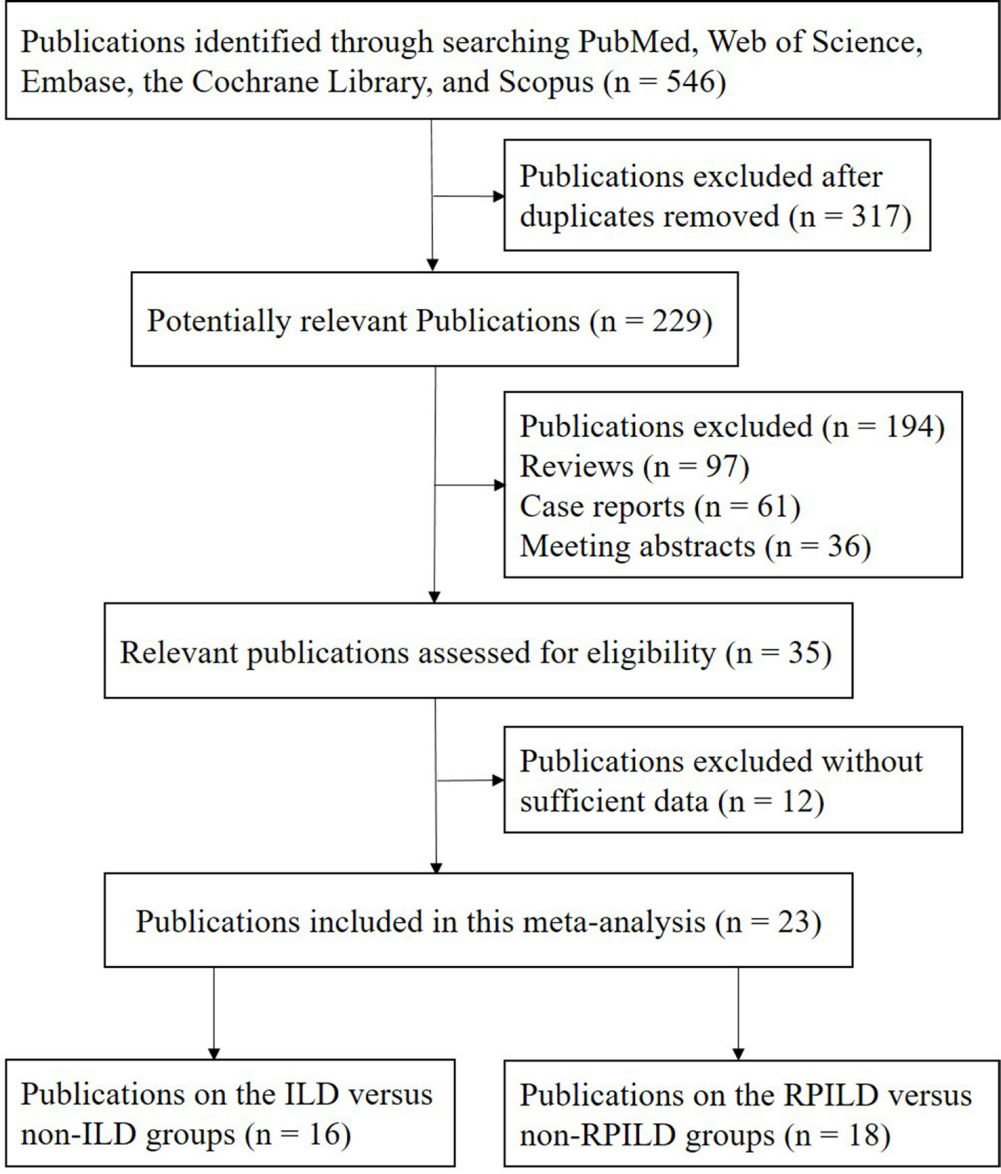
Flow diagram of publications selection procedure

The study selection process is shown in Figure 1. Initially, a total of 546 relevant studies were identified from PubMed, Web of Science, Embase, the Cochrane Library, and Scopus databases. According to the inclusion criteria, 23 publications were included, consisting of eleven publications [12, 14, 16–24] investigating the association between anti-MDA5 antibody and DM-ILD, as well as the association between anti-MDA5 antibody and DM-RPILD. Five publications [15, 25–28] investigated only the association between anti-MDA5 antibody and DM-ILD, while seven publications [29–35] investigated only the association between anti-MDA5 antibody and DM-RPILD. Therefore, 16 publications [12, 14–28] analyzing the correlation between anti-MDA5 antibody and DM-ILD and 18 publications [12, 14, 16–24, 29–35] analyzing the correlation between anti-MDA5 antibody and DM-RPILD were included in the final analysis. The main characteristics of the eligible studies are presented in Supplementary Tables 1 and 2.

**Table 1:** The summary of OR in DM with ILD versus DM without ILD

	Publications	ILD cases	Non-ILD cases	*I*^2^ (%)	*P*	Overall OR	95% CI	*P*
Total	16	491	605	0.0	0.804	16.47	10.16–26.70	< 0.001
Subgroup								
Age								
Adult	12	282	320	0.0	0.997	10.50	5.86–18.83	< 0.001
Juvenile	2	28	29	0.0	0.594	119.29	13.15–1081.93	< 0.001
Ethnicity								
Asian	12	443	427	0.0	0.871	21.25	11.47–39.34	< 0.001
European	2	8	37	0.0	0.967	9.61	1.60–57.62	0.013
Method								
Immunoprecipitation	11	377	535	0.0	0.560	15.48	9.18–26.12	< 0.001
ELISA	5	114	70	0.0	0.896	22.17	6.25–78.65	< 0.001

OR, odds ratio; DM, dermatomyositis; ILD, interstitial lung disease; ELISA, enzyme-linked immunosorbent assay.

**Table 2:** The summary of OR in DM with RPILD versus DM without RPILD

	Publications	RPILD cases	Non-RPILD cases	*I^2^* (%)	*P*	Overall OR	95% CI	*P*
Total	18	186	790	0.0	0.679	25.33	16.02–40.05	< 0.001
Subgroup								
Age								
Adult	16	175	744	0.0	0.552	24.82	15.55–39.61	< 0.001
Juvenile	2	11	46	0.0	0.790	34.84	3.88–312.62	0.002
Ethnicity								
Asian	16	170	612	0.0	0.763	26.29	16.00–43.20	< 0.001
Method								
Immunoprecipitation	11	95	423	0.0	0.741	20.31	11.03–37.39	< 0.001
ELISA	7	80	261	3.3	0.401	31.86	14.82–68.46	< 0.001

OR, odds ratio; DM, dermatomyositis; RPILD, rapidly progressive interstitial lung disease; ELISA, enzyme-linked immunosorbent assay.

### Association between anti-MDA5 antibody and ILD risk of DM patients

**Figure 2:**
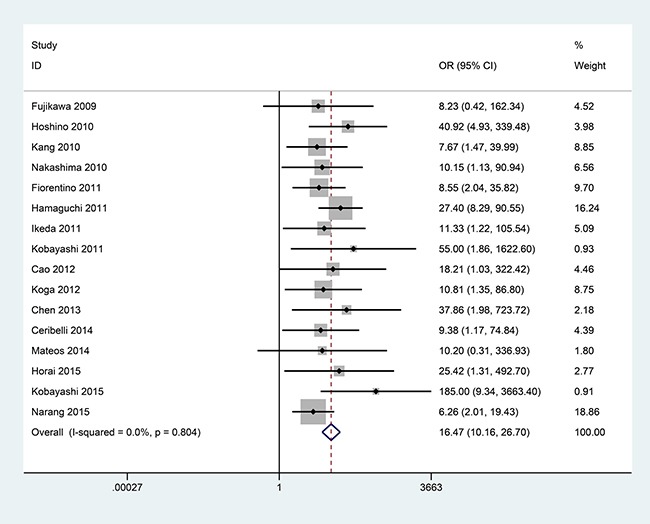
Forest plot of the association between anti-MDA5 antibody and ILD risk of DM patients with 491 DM with ILD versus 605 DM without ILD

When 491 DM with ILD were compared to 605 DM without ILD, no substantial heterogeneity was observed in the analysis with 16 publications (*I^2^* = 0.0%, *P* = 0.804, Figure 2). Thus, a fixed-effects model was used to calculate the pooled odds ratio (OR). The results revealed that a significant association was observed between the frequency of anti-MDA5 antibody and ILD of DM patients (OR = 16.47, 95% CI: 10.16–26.70, *P* < 0.001, Figure 2).

### Subgroup analysis of anti-MDA5 antibody and ILD risk of DM patients

The subgroup analyses were performed according to age (adult, juvenile), ethnicity (Asian, European), and detection method (immunoprecipitation, enzyme-linked immunosorbent assay (ELISA)) (Table 1). When using a fixed-effects model to calculate the overall ORs, no substantial heterogeneity was observed (*I^2^* < 50%, *P* > 0.10, Supplementary Figures 1–6).

In the subgroup analysis based on age, twelve publications of 282 adult DM with ILD versus 320 adult DM without ILD, as well as two publications of 28 juvenile DM with ILD versus 29 juvenile DM without ILD were examined. The pooled OR was 10.50 (95% CI: 5.86–18.83, *P* < 0.001) for the adult subgroup and 119.29 (95% CI: 13.15–1081.93, *P* < 0.001) for the juvenile subgroup.

When a subgroup analysis was performed based on ethnicity, the overall OR from twelve publications of 443 Asian DM with ILD versus 427 Asian DM without ILD was 21.25 (95% CI: 11.47–39.34, *P* < 0.001), and the overall OR from two publications of 8 European DM with ILD versus 37 DM without ILD was 9.61 (95% CI: 1.60–57.62, *P* = 0.013).

The subgroup analysis conducted according to detection method involved eleven publications of 377 DM with ILD versus 535 DM without ILD using the immunoprecipitation method, as well as five publications of 114 DM with ILD versus 70 DM without ILD using the ELISA method. Anti-MDA5 antibody was associated with the ILD of DM patients with the immunoprecipitation method (OR = 15.48, 95% CI: 9.18–26.12, *P* < 0.001), as well as with the ELISA method (OR = 22.17, 95% CI: 6.25–78.65, *P* < 0.001).

### Association between anti-MDA5 antibody and RPILD risk in DM patients

**Figure 3:**
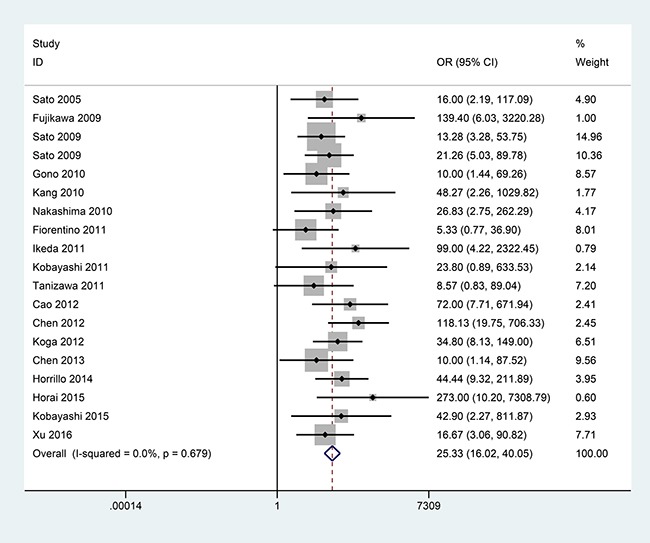
Forest plot of the association between anti-MDA5 antibody and RPILD risk of DM patients with 186 DM with RPILD versus 790 DM without RPILD

In the comparison of 186 DM with RPILD and 790 DM without RPILD from 18 publications, no substantial heterogeneity was observed (*I^2^* = 0.0%, *P* = 0.679, Figure 3). The pooled OR showed that the presence of anti-MDA5 antibody was significantly higher in DM with RPILD than DM without RPILD (OR = 25.33, 95% CI: 16.02–40.05, *P* < 0.001) (Figure 3).

### Subgroup analysis of anti-MDA5 antibody and RPILD risk in DM patients

The subgroup analyses were conducted stratified by age (adult, juvenile), ethnicity (Asian), and detection method (immunoprecipitation, ELISA) (Table 2). No substantial heterogeneity was observed when the pooled ORs were calculated using a fixed-effects model (*I^2^* < 50%, *P* > 0.10, Supplementary Figures 7–11).

In the subgroup analysis by age, sixteen publications of 175 adult DM with RPILD versus 744 adult DM without RPILD, as well as two publications of 11 juvenile DM with RPILD versus 46 juvenile DM without RPILD were examined. The pooled OR showed that the frequency of anti-MDA5 antibody in DM with RPILD was significantly higher than in DM without RPILD in the adult subgroup (OR = 24.82, 95% CI: 15.55–39.61, *P* < 0.001), as well as in the juvenile subgroup (OR = 34.84, 95% CI: 3.88–312.62, *P* = 0.002).

When a subgroup analysis was performed based on ethnicity, the pooled OR from sixteen publications involving 170 Asian DM with ILD and 612 Asian DM without ILD was 26.29 (95% CI: 16.00–43.20, *P* < 0.001).

One publication [34] detected the frequency of anti-MDA5 antibody in 17 DM with RPILD and 50 DM without RPILD using immunoprecipitation and ELISA assays. According to the detection method, the overall OR showed that the presence of anti-MDA5 antibody was significantly associated with RPILD risk in DM patients in immunoprecipitation assays that compared 95 DM with RPILD and 423 DM without RPILD from eleven publications (OR = 20.31, 95% CI: 11.03–37.39, *P* < 0.001), as well as in ELISA assays that compared 80 DM with RPILD and 261 DM without RPILD from seven publications (OR = 31.86, 95% CI: 14.82–68.46, *P* < 0.001).

### Diagnostic capacity of anti-MDA5 antibody for ILD of DM patients

**Figure 4:**
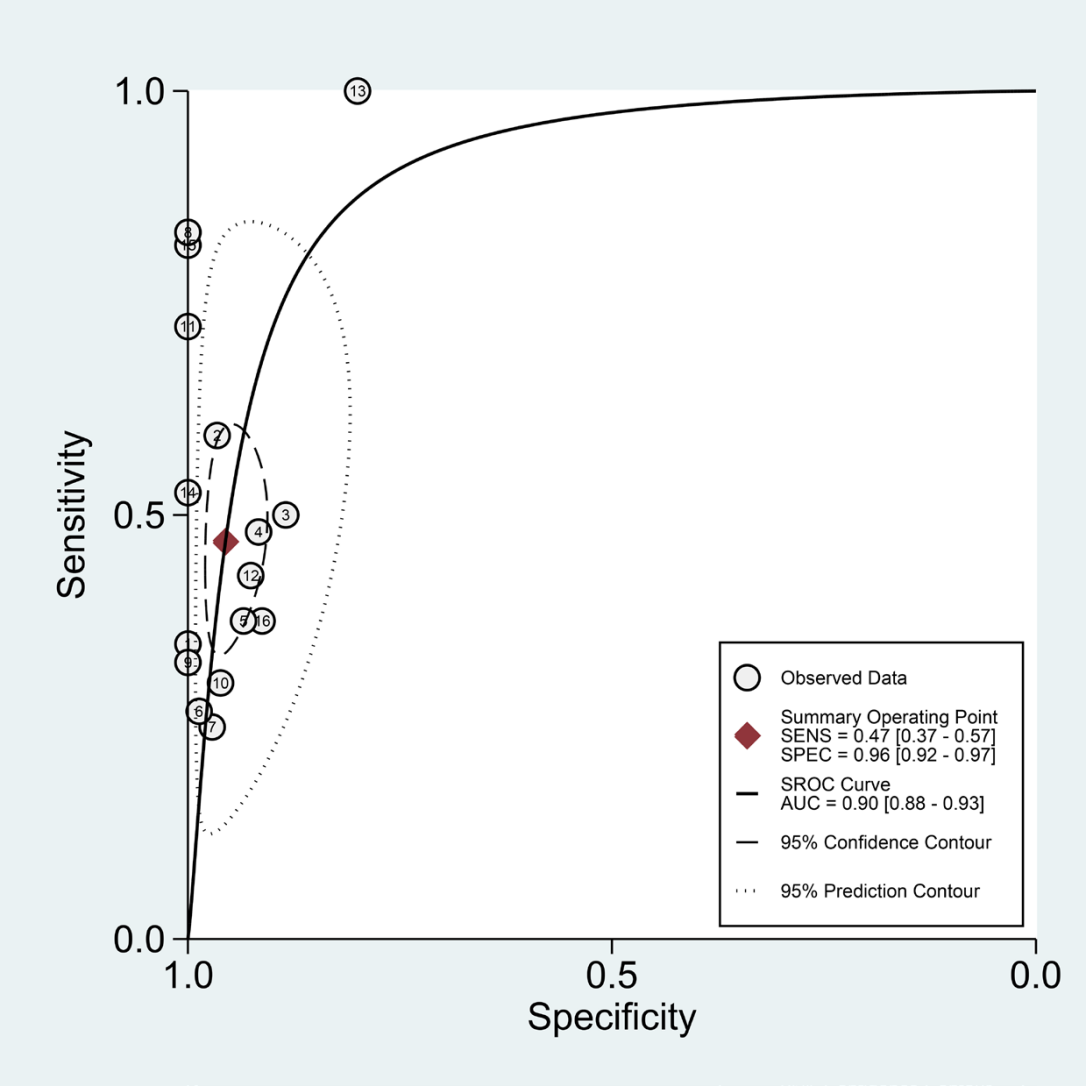
The SROC of the accuracy of anti-MDA5 antibody in the diagnosis of ILD in DM patients

The pooled sensitivity, specificity and area under the curve of the summary receiver operating characteristic (AUC) values of anti-MDA5 antibody for DM with ILD versus without ILD were 0.47 (95% CI: 0.37–0.57), 0.96 (95% CI, 0.92–0.97), and 0.90 (95% CI: 0.88–0.93) (Figure 4), respectively. The outcomes demonstrated that anti-MDA5 antibody had a low diagnostic accuracy for ILD in DM patients.

### Subgroup analysis of diagnostic capacity of anti-MDA5 antibody for ILD of DM patients

The diagnostic accuracy of anti-MDA5 antibody for DM with ILD versus without ILD was calculated in the subgroup analyses by age (adult), ethnicity (Asian), and testing methods (immunoprecipitation, ELISA) (Table 3).

**Table 3:** Diagnostic capacity of anti-MDA5 antibody in ILD of DM patients

	Pooled sensitivity	95% CI	Pooled specificity	95% CI	AUC	95% CI
Total	0.47	0.37–0.57	0.96	0.92–0.97	0.90	0.88–0.93
Subgroup						
Age						
Adult	0.42	0.34–0.52	0.93	0.90–0.96	0.91	0.88–0.93
Ethnicity						
Asian	0.48	0.36–0.60	0.97	0.95–0.99	0.97	0.95–0.98
Method						
Immunoprecipitation	0.43	0.33–0.55	0.95	0.92–0.97	0.87	0.84–0.90
ELISA	0.55	0.37–0.72	0.97	0.73–1.00	0.97	0.95–0.98

MDA5, melanoma differentiation associated gene 5; ILD, interstitial lung disease; DM, dermatomyositis; CI, confidence interval; AUC, area under the curve of the summary receiver operating characteristic; ELISA, enzyme-linked immunosorbent assay.

The pooled sensitivity, specificity, and AUC values, respectively, were 0.42 (95% CI: 0.34–0.52), 0.93 (95% CI, 0.90–0.96), and 0.91 (95% CI: 0.88–0.93) for the adult subgroup, and were 0.48 (95% CI: 0.36–0.60), 0.97 (95% CI, 0.95–0.99), and 0.97 (95% CI: 0.95–0.98) for the Asian subgroup.

In addition, a subgroup analysis was performed according to the testing methods. When anti-MDA5 antibody was detected using the immunoprecipitation method, the pooled sensitivity, specificity, and AUC were 0.43 (95% CI: 0.33–0.55), 0.95 (95% CI, 0.92–0.97), and 0.87 (95% CI: 0.84–0.90), respectively. When anti-MDA5 antibody was detected using the ELISA method, the pooled sensitivity, specificity, and AUC were 0.55 (95% CI: 0.37–0.72), 0.97 (95% CI, 0.73–1.00), and 0.97 (95% CI: 0.95–0.98), respectively.

The results in all subgroup analyses suggested that the presence of anti-MDA5 antibody had a low diagnostic value for ILD in DM patients.

### Diagnostic capacity of anti-MDA5 antibody for RPILD of DM patients

**Figure 5:**
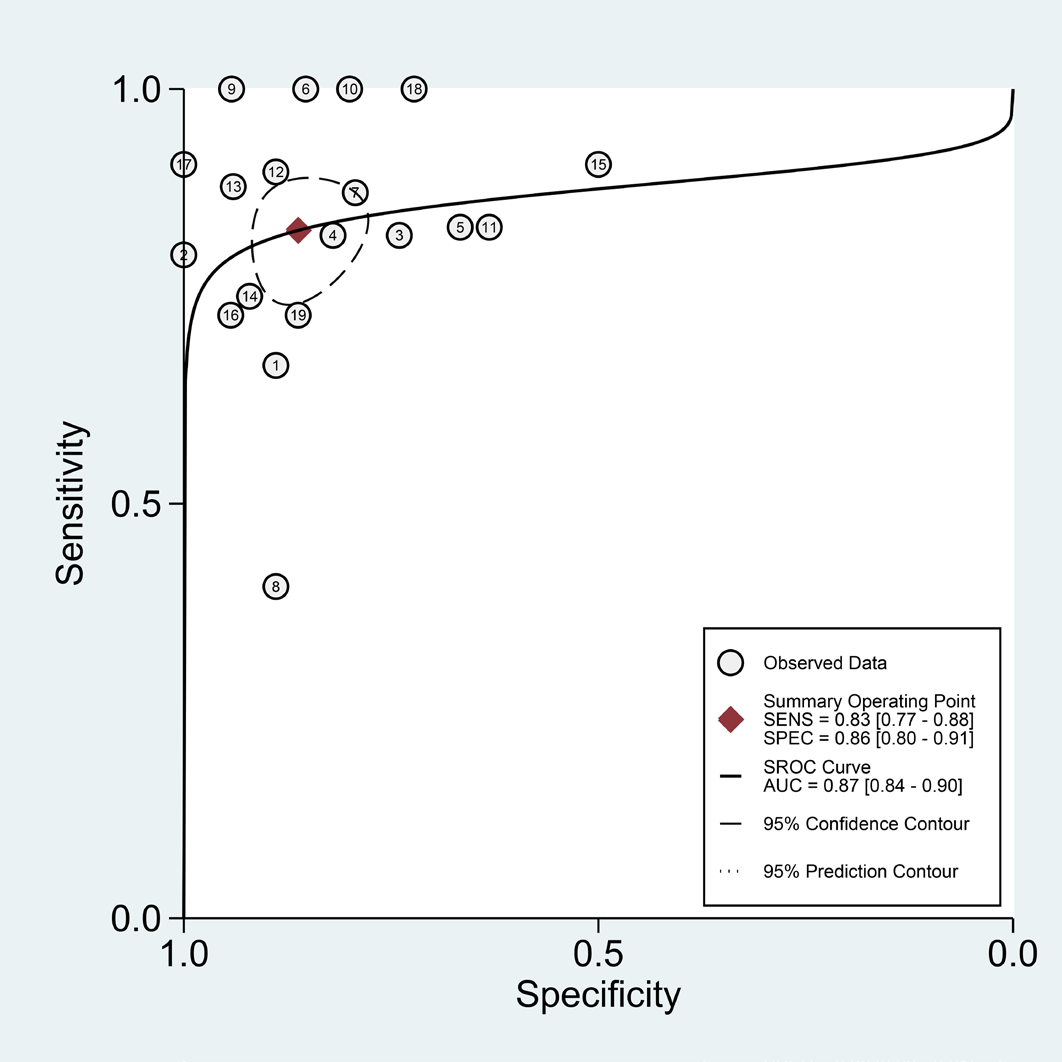
The SROC of the accuracy of anti-MDA5 antibody in the diagnosis of RPILD in DM patients

The overall sensitivity, specificity and AUC values for anti-MDA5 antibody in DM with RPILD versus without RPILD were 0.83 (95% CI: 0.77–0.88), 0.86 (95% CI, 0.80–0.91), and 0.87 (95% CI: 0.84–0.90) (Figure 5), respectively. The results demonstrated that the detection of anti-MDA5 antibody provided good diagnostic accuracy for RPILD in DM patients.

### Subgroup analysis of diagnostic capacity of anti-MDA5 antibody for RPILD of DM patients

The subgroup analyses were performed according to age (adult), ethnicity (Asian), and detection method (immunoprecipitation, ELISA) (Table 4).

**Table 4:** Diagnostic capacity of anti-MDA5 antibody in RPILD of DM patients

	Pooled sensitivity	95% CI	Pooled specificity	95% CI	AUC	95% CI
Total	0.83	0.77–0.88	0.86	0.80–0.91	0.87	0.84–0.90
Subgroup						
Age						
Adult	0.82	0.75–0.87	0.87	0.81–0.91	0.85	0.81–0.88
Ethnicity						
Asian	0.85	0.78–0.89	0.85	0.78–0.90	0.87	0.84–0.90
Method						
Immunoprecipitation	0.81	0.71–0.88	0.85	0.78–0.90	0.88	0.85–0.91
ELISA	0.86	0.77–0.92	0.86	0.74–0.94	0.87	0.84–0.90

MDA5, melanoma differentiation associated gene 5; RPILD, rapidly progressive interstitial lung disease; DM, dermatomyositis; CI, confidence interval; AUC, area under the curve of the summary receiver operating characteristic; ELISA, enzyme-linked immunosorbent assay.

The pooled sensitivity, specificity, and AUC values of anti-MDA5 antibody, respectively, were 0.82 (95% CI: 0.75–0.87), 0.87 (95% CI, 0.81–0.91), and 0.85 (95% CI: 0.81–0.88) in adult DM with RPILD versus without RPILD, and were 0.85 (95% CI: 0.78–0.89), 0.85 (95% CI, 0.78–0.90), and 0.87 (95% CI: 0.84–0.90) for diagnosing RPILD in Asian DM patients.

In the stratified analysis by testing methods, the overall sensitivity, specificity, and AUC values for anti-MDA5 antibody in DM with RPILD versus without RPILD were 0.81 (95% CI: 0.71–0.88), 0.85 (95% CI, 0.78–0.90), and 0.88 (95% CI: 0.85–0.91) with immunoprecipitation, and were 0.86 (95% CI: 0.77–0.92), 0.86 (95% CI, 0.74–0.94), and 0.87 (95% CI: 0.84–0.90) with ELISA.

The results of all subgroups showed that anti-MDA5 antibody had good diagnostic value for RPILD in DM patients.

### Trial sequential analysis and statistical power

Trial sequential analysis (TSA) was conducted with a type I error of 5% and type II error of 20% (power of 80%). The control event proportions were calculated from the included studies, and there was a relative risk reduction (RRR) of 35% for DM with ILD versus without ILD, while a RRR of 65% was found for DM with RPILD versus without RPILD. The cumulative Z curve was calculated with a fixed-effects model.

When TSA for DM with ILD versus without ILD was performed, six publications [14, 17, 20, 24, 27, 31] were ignored in interim looks due to too low information use (< 1%). The TSA result showed that the cumulative Z-curve crossed both the conventional boundary and the trial sequential monitoring boundary (Supplementary Figure 12). There was sufficient evidence to support that a higher frequency of anti-MDA5 antibody was in DM with ILD compared with DM without ILD, and no further trials were needed.

The outcome of TSA for DM with RPILD versus without RPILD revealed that the Z-curve crossed the trial sequential monitoring boundary and reached the required information size (Supplementary Figure 13), with sufficient evidence that anti-MDA5 antibody was more associated with DM with RPILD rather than DM without RPILD, and no further trials were required.

The results of the statistical power calculation showed the sample size of the meta-analysis for DM with ILD versus that without ILD had more than 99% power (α = 0.05) when the positive rates of anti-MDA5 antibody for DM with ILD and DM without ILD were defined as 40.53% and 4.13%, respectively (Supplementary Figure 14). For the meta-analysis for DM with RPILD versus without RPILD, the actual power was more than 99% (α = 0.05) when the positive rates of anti-MDA5 antibody for DM-RPILD and DM without RPILD were defined as 82.80% and 14.18%, respectively (Supplementary Figure 15).

### Publication bias

The analysis of publication bias was performed using Egger's test in DM with ILD versus without ILD (*P* = 0.121, Supplementary Figure 16), as well as in DM with RPILD versus without RPILD (*P* = 0.173, Supplementary Figure 17). The results revealed no evidence of publication bias (*P* > 0.05).

## DISCUSSION

A recent study found that anti-MDA5 antibody is associated with DM and not with polymyositis [36]. ILD is considered a life-threatening complication in patients with DM, which can develop RPILD and have fatal consequences [37]. Early diagnosis and aggressive therapy are required in DM-ILD and DM-RPILD to improve poor prognosis [5, 7]. Anti-MDA5 antibody has been assessed in the ILD or RPILD of DM patients [14–17]. However, results regarding an association between the frequency of anti-MDA5 antibody and the pulmonary involvements in patients with DM have been inconsistent; additionally, the diagnostic values of anti-MDA5 antibody for DM-ILD and DM-RPILD remain confusing. Consequently, we performed the current meta-analysis to assess the values of anti-MDA5 antibody for ILD or RPILD in DM patients.

The current meta-analysis comprehensively analyzed published data from sixteen publications with 491 DM with ILD versus 605 DM without ILD, as well as eighteen publications with 186 DM with RPILD versus 790 DM without RPILD. The results comprising multiple studies with a large sample size revealed that the presence of anti-MDA5 antibody was significantly associated with DM-ILD and DM-RPILD, which was confirmed by TSA. Additionally, the subgroup analyses by age, ethnicity, and testing methods were carried out to determine the differences in various subgroups.

The subgroup analysis based on age showed that anti-MDA5 antibody was more associated with DM-ILD in the juvenile subgroup than in the adult subgroup (OR: 119.29 vs. 10.50). The results revealed that juvenile patients may be more susceptible to anti-MDA5 antibody than adult patients. When comparing the association between anti-MDA5 antibody and ILD with the association between anti-MDA5 antibody and RPILD, we found that anti-MDA5 antibody was more associated with RPILD than ILD in the adult subset (OR: 24.82 vs. 10.50); conversely, in the juvenile subset, ILD was more frequent than RPILD (OR: 119.29 vs. 34.84). These outcomes indicated that anti-MDA5-positive adult DM patients have a higher risk of developing RPILD, while anti-MDA5-positive juvenile DM patients may have a higher risk of developing ILD. The appearance of anti-MDA5 antibody at different ages may suggest the disease progression of DM patients. However, the result should be interpreted with caution due to the small sample size of juvenile patients examined.

In the subgroup analysis based on ethnicity, DM with ILD in the Asian population showed that anti-MDA5 antibody was more frequent than those in the European population (OR: 21.25 vs. 9.61). The results showed that the positive rate of anti-MDA5 antibody varies between ethnic populations. Asian DM-ILD patients were more likely than European DM-ILD patients to develop anti-MDA5 antibody. When comparing the association between anti-MDA5 antibody and ILD with the association between anti-MDA5 antibody and RPILD, the correlations between anti-MDA5 antibody with ILD and RPILD were similar in the Asian subgroup (OR: 21.25 vs. 26.29). The outcomes suggested that the risk for developing ILD is nearly equal to that for developing RPILD in Asian DM patients. However, more studies are needed to confirm this association due to the small sample size of European patients. Additionally, studies employing European DM-RPILD patients are scarce, so the connection between anti-MDA5 antibody and the RPILD of European DM patients was not evaluated.

In the subgroup analysis according to testing methods, the pooled ORs in DM with ILD versus without ILD (OR: 15.48 vs. 22.17), as well as in DM with RPILD versus without RPILD (OR: 20.31 vs. 31.86), were similar between the immunoprecipitation and ELISA methods. In comparing the association between anti-MDA5 antibody with ILD with the association between anti-MDA5 antibody with RPILD, the pooled ORs were similar when using immunoprecipitation (OR: 15.48 vs. 20.31), as well as using ELISA (OR: 22.17 vs. 31.86). These results suggested that both immunoprecipitation and ELISA were sensitive to detect anti-MDA5 antibody.

The results demonstrated that a significant relationship was found between anti-MDA5 antibody and ILD or RPILD in DM patients, indicating that anti-MDA5 antibody has potential value as a noninvasive biomarker for DM-ILD and DM-RPILD. In addition, some researchers have reported that anti-MDA5 antibody was a useful biomarker of DM-ILD and DM-RPILD [12, 31, 38]. Hence, we further analyzed the diagnostic accuracy of anti-MDA5 antibody for DM-ILD and DM-RPILD. In the present study, the diagnostic values of anti-MDA5 antibody were evaluated in DM with ILD versus without ILD, as well as in DM with RPILD versus without RPILD. The results suggested that anti-MDA5 antibody has low diagnostic value in the diagnosis of DM-ILD, with high specificity (0.96) but relatively low sensitivity (0.47). Likewise, the subgroup analyses according to age, ethnicity, and detection methods showed similar results (sensitivity < 0.6), indicating that anti-MDA5 antibody could not distinguish between DM with ILD and DM without ILD. Notably, anti-MDA5 antibody was a valuable biomarker for identifying the risk of RPILD in patients with DM with good sensitivity (0.83) and good specificity (0.86). Similarly, the outcomes of subgroup analyses revealed that anti-MDA5 antibody had a good diagnostic accuracy for RPILD in DM patients (sensitivity > 0.80, specificity > 0.80). Taken together, the presence of anti-MDA5 antibody is a more appropriate screening index for RPILD rather than ILD in patients with DM.

This meta-analysis has some limitations. First, only published English language literature was included. Studies in other languages were excluded due to insufficient information, which may lead to selection bias. Second, due to the small sample size of juvenile and European patients, the association between anti-MDA5 antibody and DM-ILD and DM-RPILD in the juvenile subgroup and in the European subgroup require additional studies to confirm our results. Third, published data regarding the juvenile and European patients were insufficient; thus, evaluations of the values of anti-MDA5 antibody in the diagnosis of DM-ILD or DM-RPILD in these two subgroups were not conducted. Additionally, the population was predominantly Asian in the current study, whereas only a small number of Caucasian and African patients were studied. Finally, the prognostic significance of anti-MDA5 antibody for DM-ILD and DM-RPILD was not assessed.

In conclusion, detecting anti-MDA5 antibody is more valuable in diagnosing RPILD than ILD in patients with DM. Well-designed prospective studies are warranted to verify the present results and evaluate the prognostic role of anti-MDA5 antibodies in DM-ILD and DM-RPILD.

## MATERIALS AND METHODS

### Search strategy

A literature search was systematically performed in PubMed, Web of Science, Embase, the Cochrane Library, and Scopus for the published English-language studies analyzing the diagnostic value of anti-MDA5 antibody in patients with DM. Publications were identified with the following index terms: “MDA5”, “melanoma differentiation-associated gene 5”, “CADM-140”, and “dermatomyositis” up to January 14, 2017. The reference lists of relevant articles and reviews were scanned to find additional studies.

### Inclusion and exclusion criteria

The following inclusion criteria were applied: (1) patients fulfilled the Bohan and Peter criteria [2, 3], Sontheimer criteria [39], or the criteria of the ENMC workshop [1]; (2) the diagnosis of ILD was established according to the results of chest radiography, chest computed tomography (CT), high-resolution chest computed tomography, and pulmonary function tests, while the RPILD was defined as a progressive deterioration of ILD within a few months from the onset of respiratory symptoms; (3) DM patients were divided into the ILD and non-ILD groups or into the RPILD and non-RPILD groups; (4) studies evaluated the frequency of anti-MDA5 antibody in the sera or plasma of patients with DM; (5) sufficient data was provided to construct a diagnostic four-fold contingency table; and (6) if the same research teams published articles with overlapping data, only the studies with the most data were included. The exclusion criteria were as follows: (1) review articles, case reports, commentaries, letters, and abstracts without sufficient information; (2) publications did not meet the inclusion criteria.

### Data extraction

A full-text review of the identified articles was independently performed by two investigators, followed by data extraction. The following information were extracted: first author, disease type, number of cases, ethnicity of patient, detection method, frequency of anti-MDA5 antibody in the ILD, non-ILD, RPILD, and non-RPILD groups. The reviewers resolved disagreements by discussion and reached a consensus.

### Statistical analysis

This meta-analysis was conducted using Stata 12.0 software (Stata Corporation, College Station, TX, USA). The pooled ORs with 95% CIs were calculated to evaluate the association between anti-MDA5 antibody and the ILD or RPILD of patients with DM. Moreover, the pooled ORs and corresponding 95% CIs were also measured to analyze the association between anti-MDA5 antibody and the ILD or RPILD in DM patients in the subgroup analyses performed according to age, ethnicity, and detection method. If *I^2^* ≥ 50% in I^2^-statistic, or the *P*-value ≤ 0.10 in Q-statistic, a heterogeneity test was performed. A bivariate mixed-effects model was used to calculate the pooled sensitivity, specificity, and AUC values to evaluate the diagnostic values of anti-MDA5 antibody for ILD or RPILD of patients with DM, as well as for ILD or RPILD of DM in the subgroup analyses stratified by age, ethnicity, and detection method [40, 41]. TSA, a useful method to determine whether sufficient and conclusive evidence has been reached in a meta-analysis [42–44], was conducted using software TSA version 0.9 beta (http://www.ctu.dk/tsa). When the cumulative Z-curve crosses the conventional boundary and the trial sequential monitoring boundary or crosses into the futility area boundary, a sufficient level of evidence may have been reached so that no further studies are needed. If the Z-curve does not cross any of the boundaries, there is insufficient evidence to reach a conclusion, and additional trials are required to confirm the outcomes. Statistical power analysis was performed using G*Power version 3.1.9.2 (Program written, concept and design by Franz, Universitat Kiel, Germany; freely available windows application software) [45] with an α level of 5%. In addition, Egger's test was performed to evaluate potential publication bias for outcomes with more than nine studies [46].

## SUPPLEMENTARY MATERIALS FIGURES AND TABLES



## Abbreviations

MDA5: melanoma differentiation-associated protein 5
DM: dermatomyositis
ILD: interstitial lung disease
RPILD: rapidly progressive interstitial lung disease
AUC: area under the curve of the summary receiver operating characteristic
TSA: trial sequential analysis
CDM: classic dermatomyositis
CADM: clinically amyopathic dermatomyositis
MSAs: myositis-specific autoantibodies
IFIH1: interferon induced with helicase C domain protein 1
OR: odds ratio
ELISA: enzyme-linked immunosorbent assay
RRR: relative risk reduction
CT: computed tomography
