# Supplementary material for: Assessment of anti-MDA5 antibody as a diagnostic biomarker in patients with dermatomyositis-associated interstitial lung disease or rapidly progressive interstitial lung disease
Source: Oncotarget. 2017 Jul 6;8(44):76129–40. doi: 10.18632/oncotarget.19050 (PMC5652692; doi:10.18632/oncotarget.19050)
Supplement: Supplementary file 1 [file oncotarget-08-76129-s001.pdf]

Assessment of anti-MDA5 antibody as a diagnostic biomarker in patients with dermatomyositis-associated interstitial lung disease or rapidly progressive interstitial lung disease

Supplementary Materials

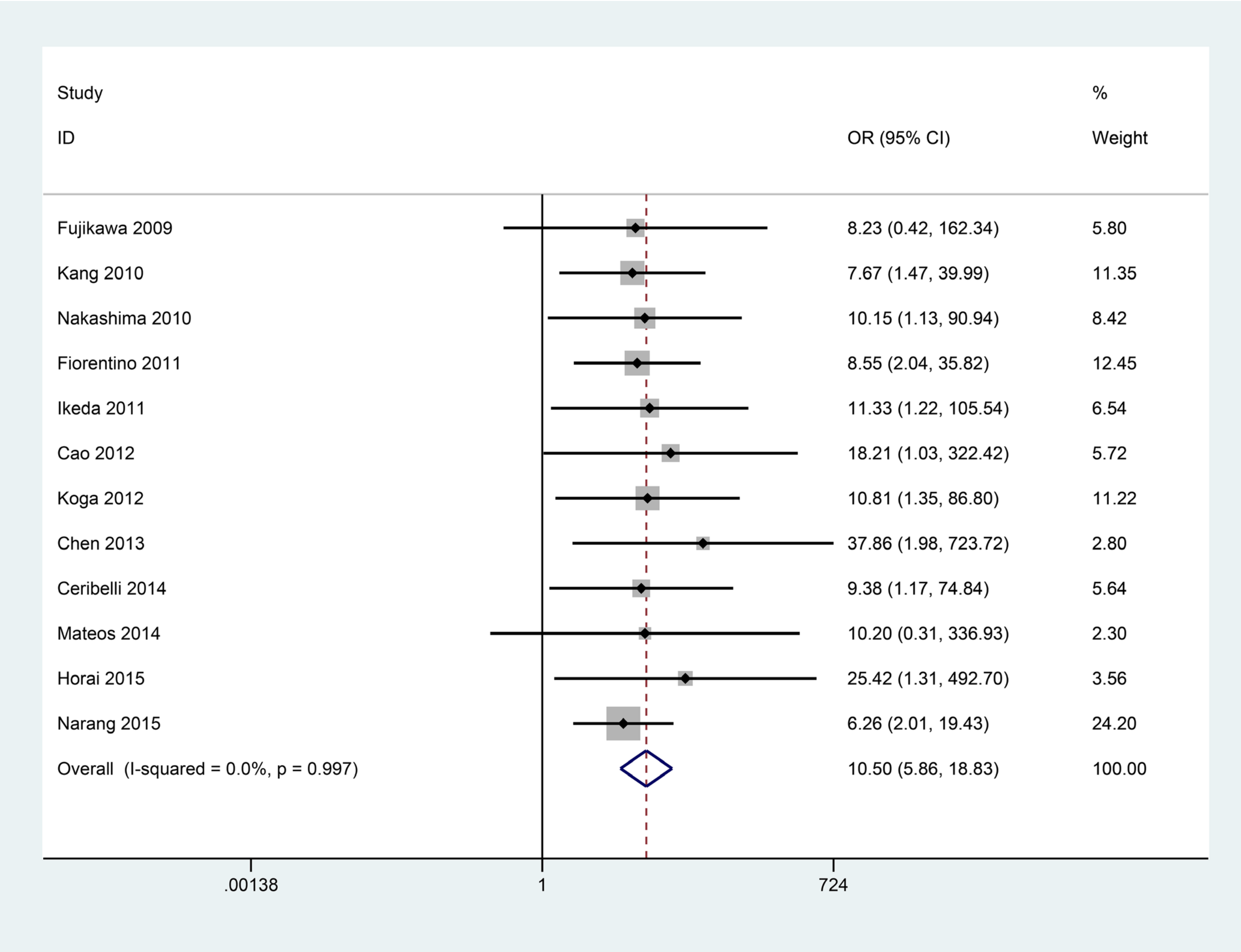

Supplementary Figure 1: Forest plot of the association between anti-MDA5 antibody and ILD risk of adult DM patients.

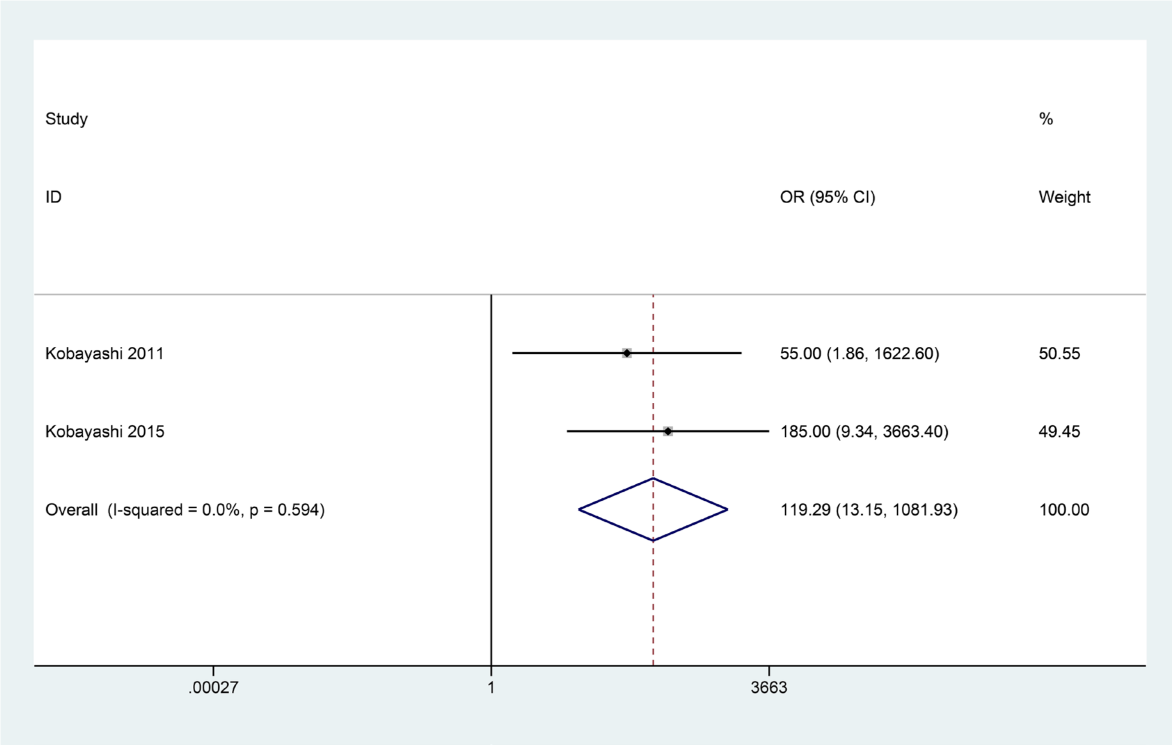

Supplementary Figure 2: Forest plot of the association between anti-MDA5 antibody and ILD risk of juvenile DM patients.

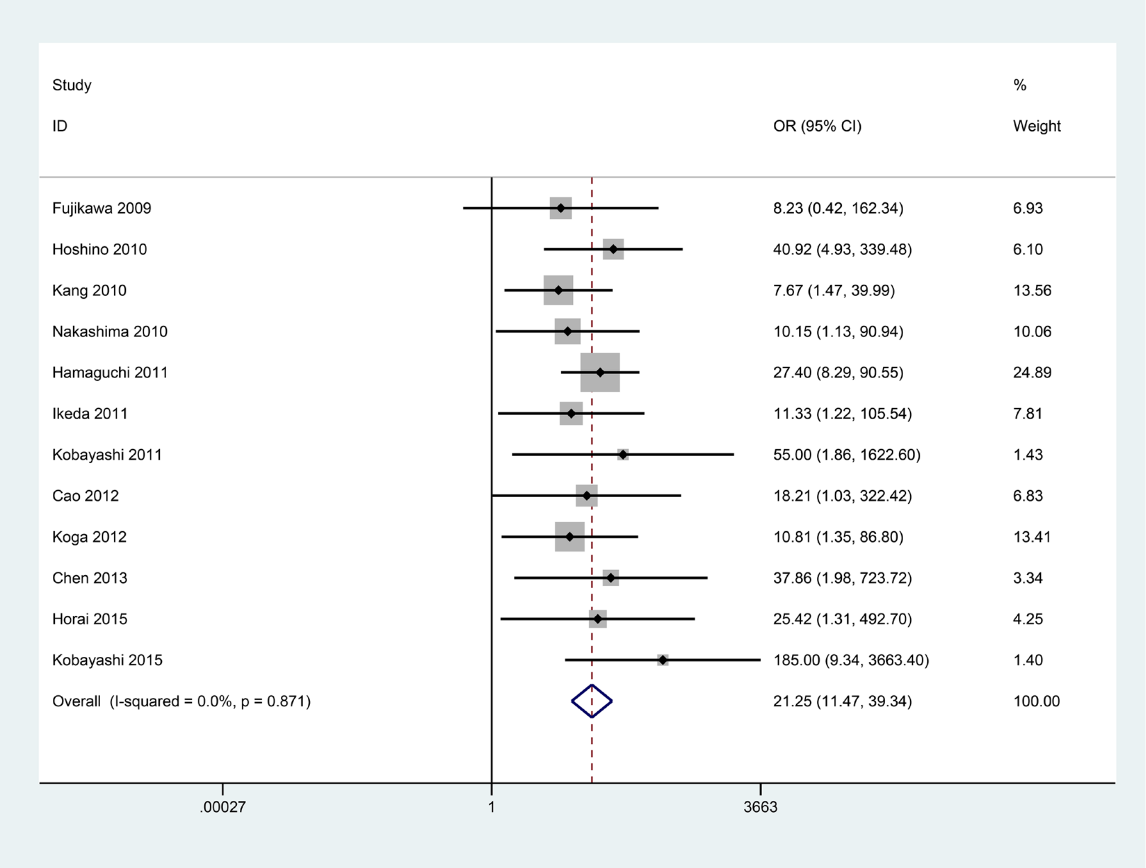

Supplementary Figure 3: Forest plot of the association between anti-MDA5 antibody and ILD risk of Asian DM patients.

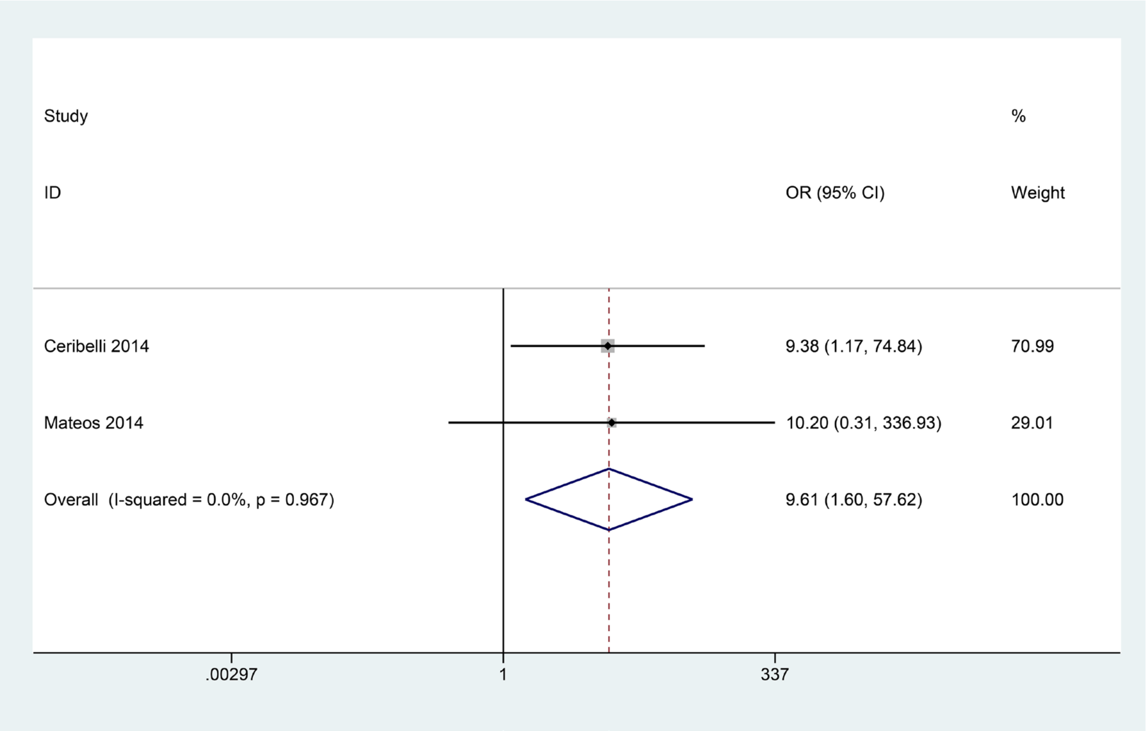

Supplementary Figure 4: Forest plot of the association between anti-MDA5 antibody and ILD risk of European DM patients.

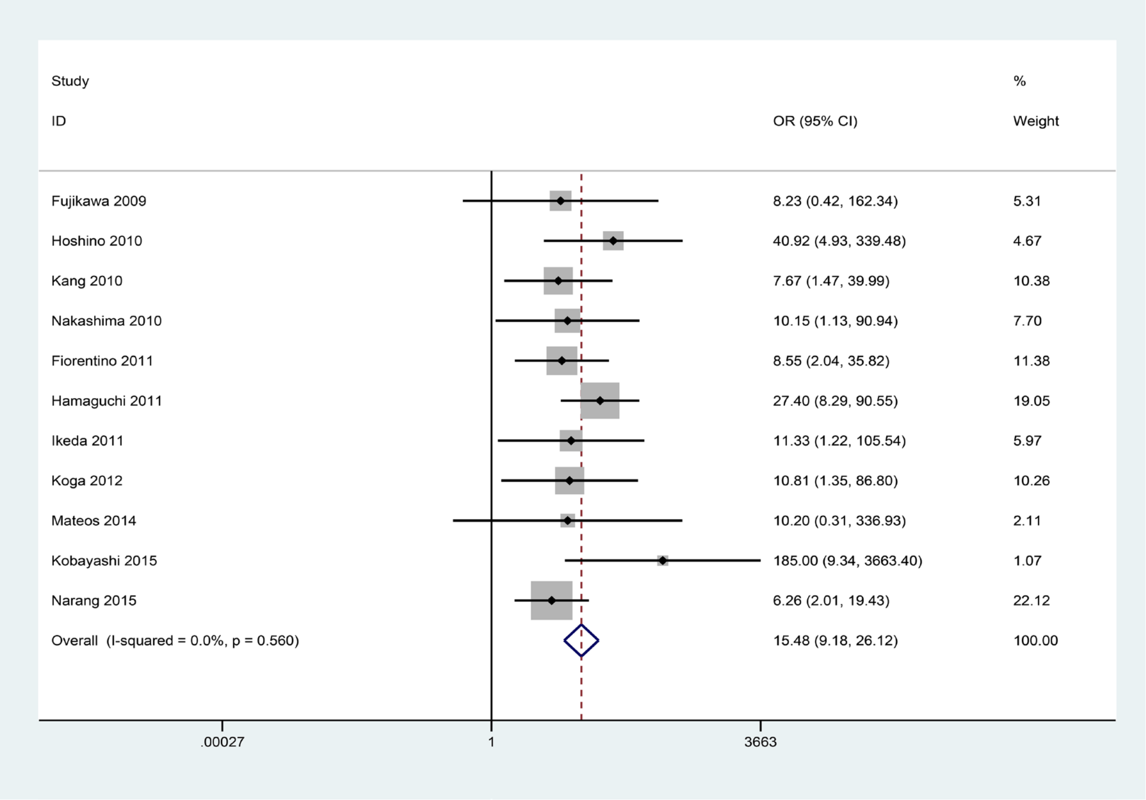

Supplementary Figure 5: Forest plot of the association between anti-MDA5 antibody and ILD risk of DM patients using immunoprecipitation.

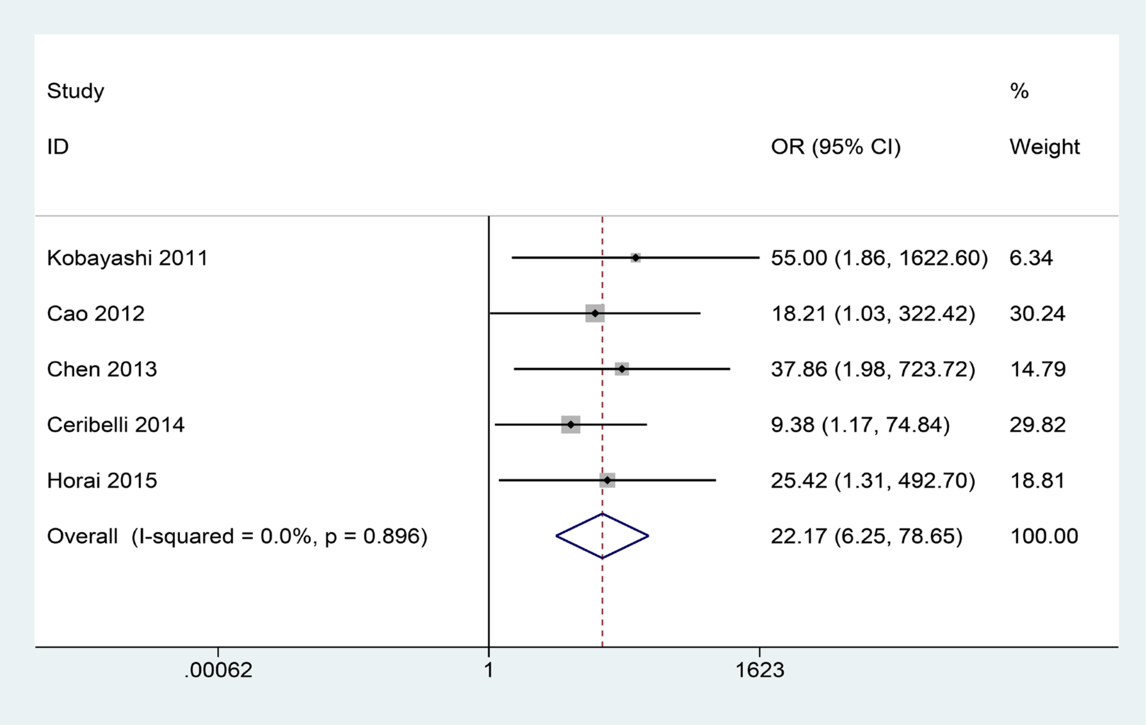

Supplementary Figure 6: Forest plot of the association between anti-MDA5 antibody and ILD risk of DM patients using ELISA.

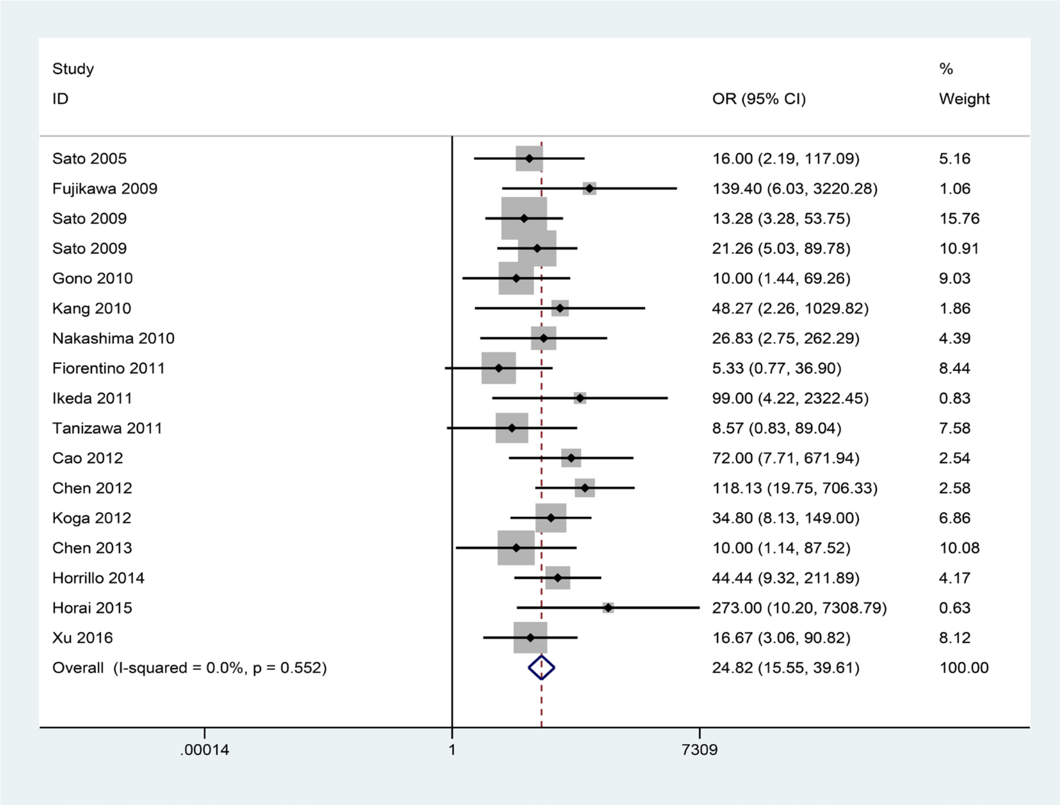

Supplementary Figure 7: Forest plot of the association between anti-MDA5 antibody and RPILD risk of adult DM patients.

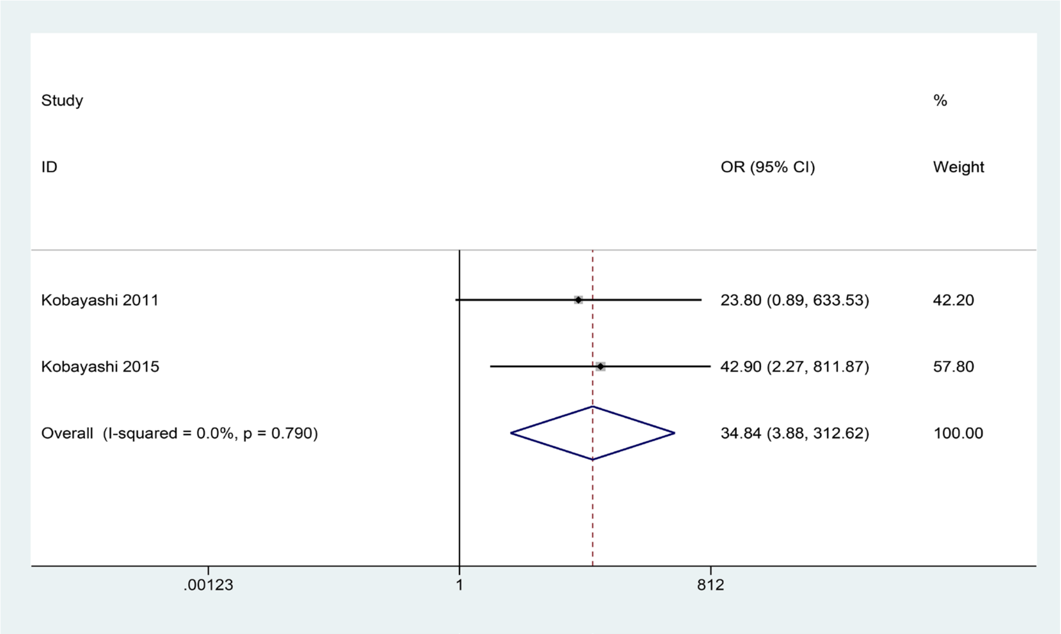

Supplementary Figure 8: Forest plot of the association between anti-MDA5 antibody and RPILD risk of juvenile DM patients.

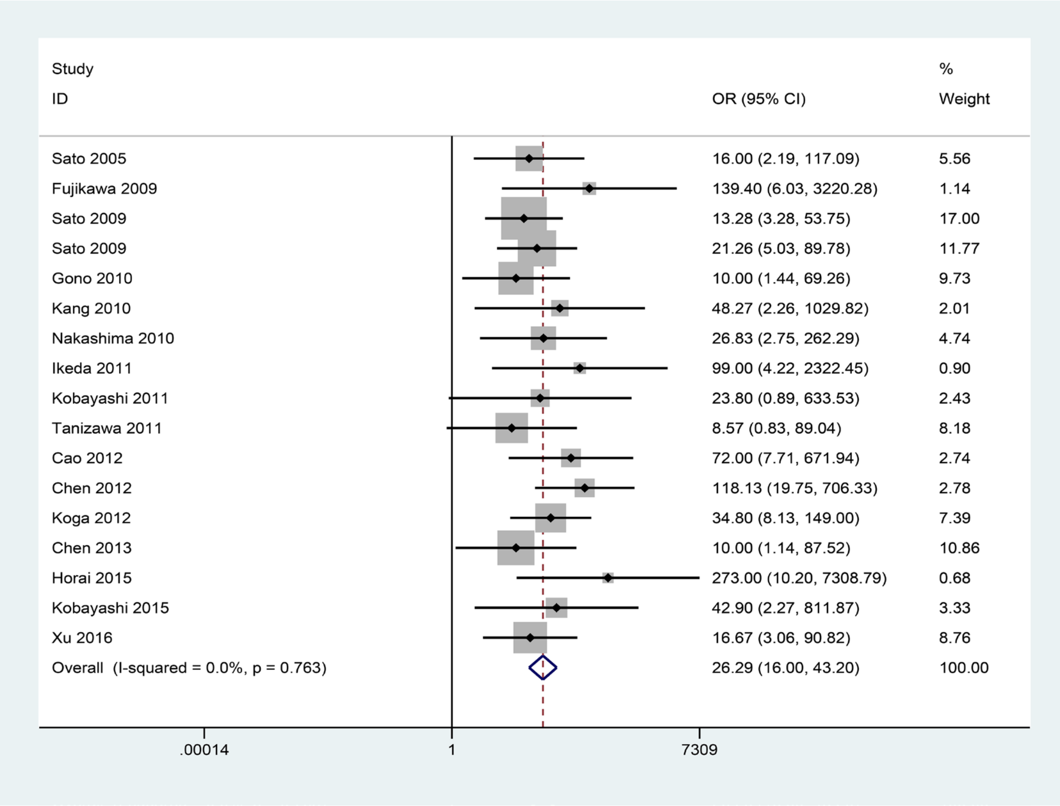

Supplementary Figure 9: Forest plot of the association between anti-MDA5 antibody and RPILD risk of Asian DM patients.

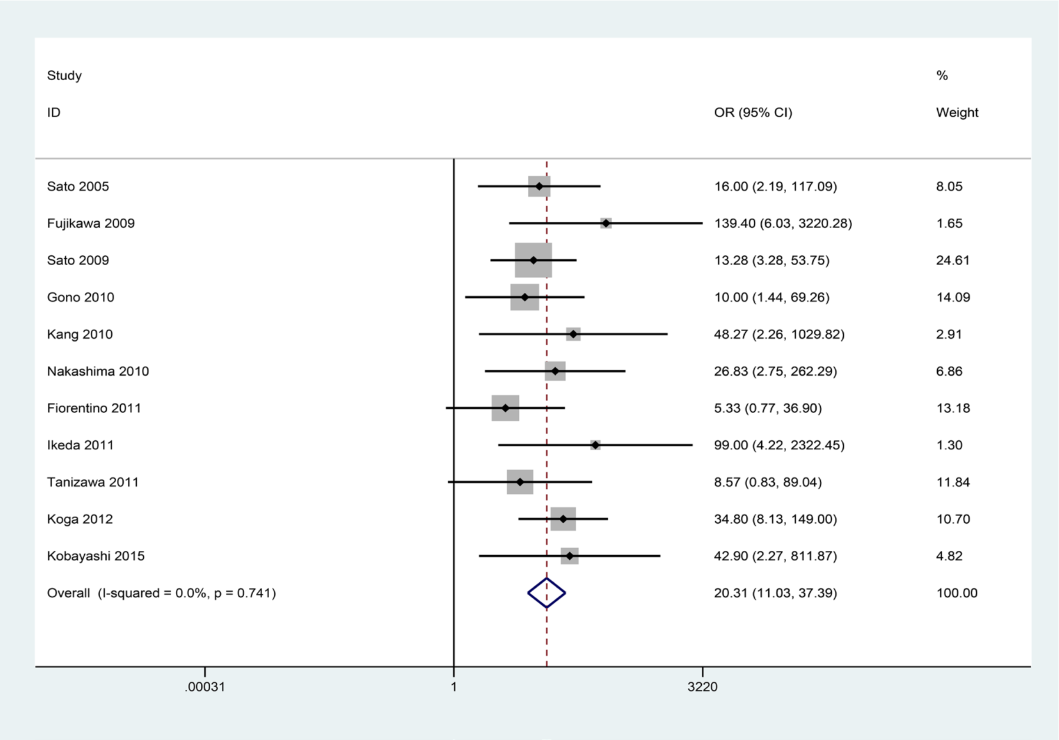

Supplementary Figure 10: Forest plot of the association between anti-MDA5 antibody and RPILD risk of DM patients using immunoprecipitation.

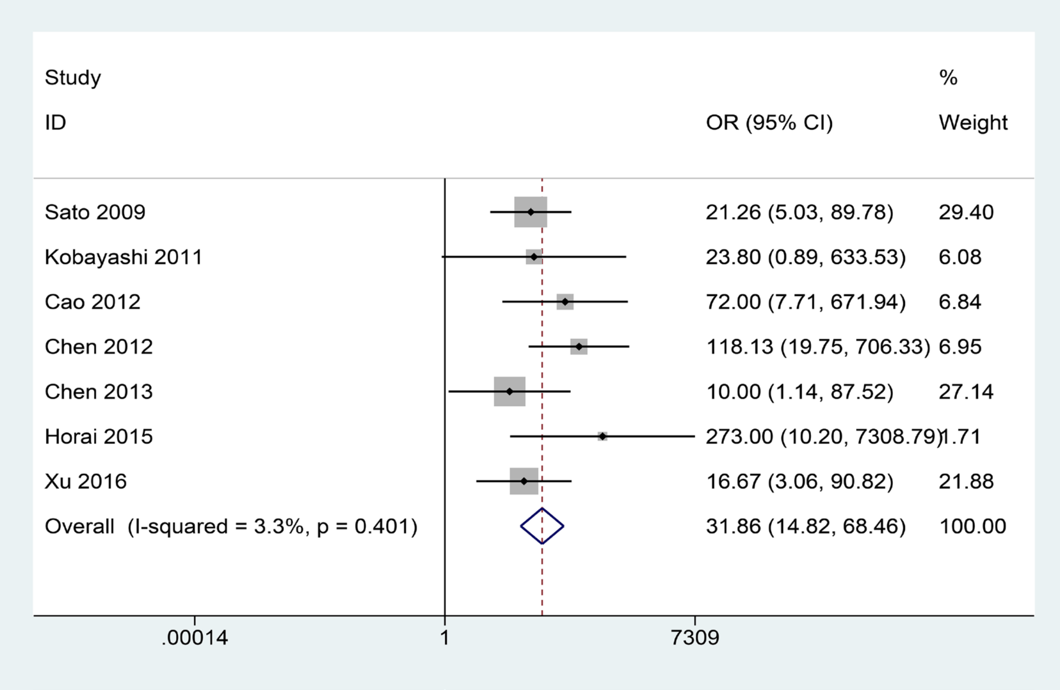

Supplementary Figure 11: Forest plot of the association between anti-MDA5 antibody and RPILD risk of DM patients using ELISA.

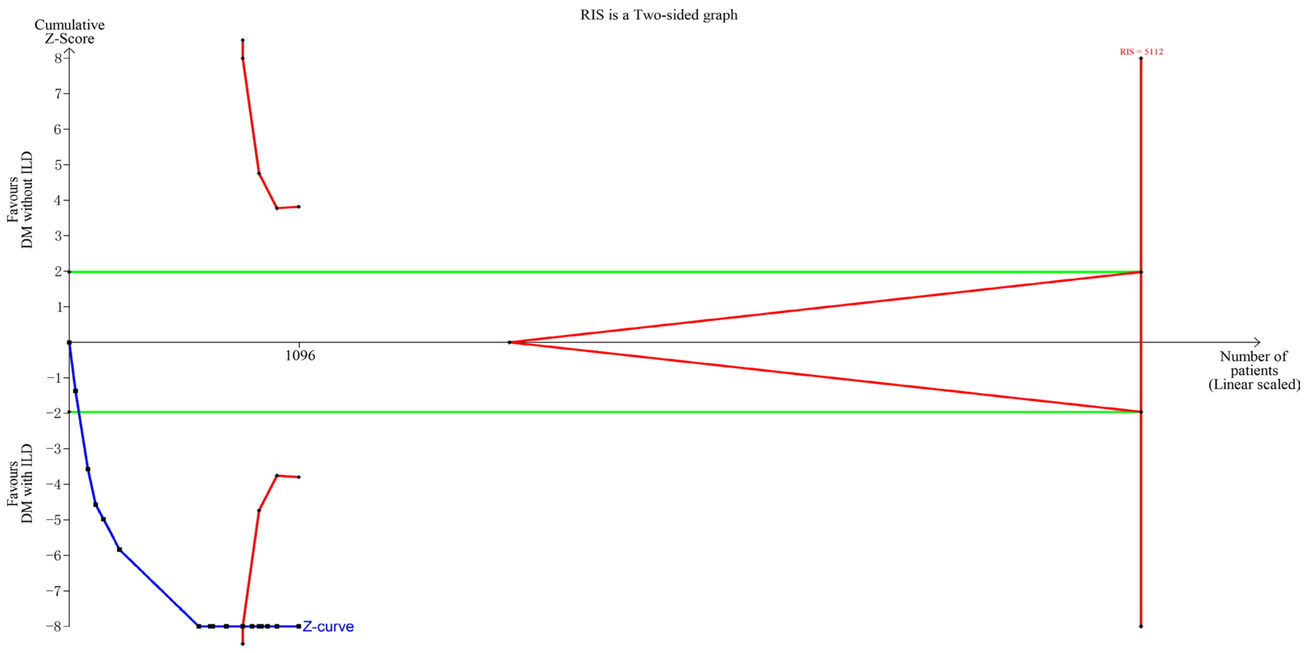

Supplementary Figure 12: Trial sequential analysis for the association between anti-MDA5 antibody and DM-ILD. A diversity adjusted information size of 5112 patients was calculated using α = 0.05 (two sided), β = 0.20 (80% of power), relative risk reduction = 35%, and an event proportion of 4% in the control arm. The complete blue line represents the cumulative Z-curve, which crossed both the conventional boundary (complete green line) and the trial sequential monitoring boundary (complete red line).

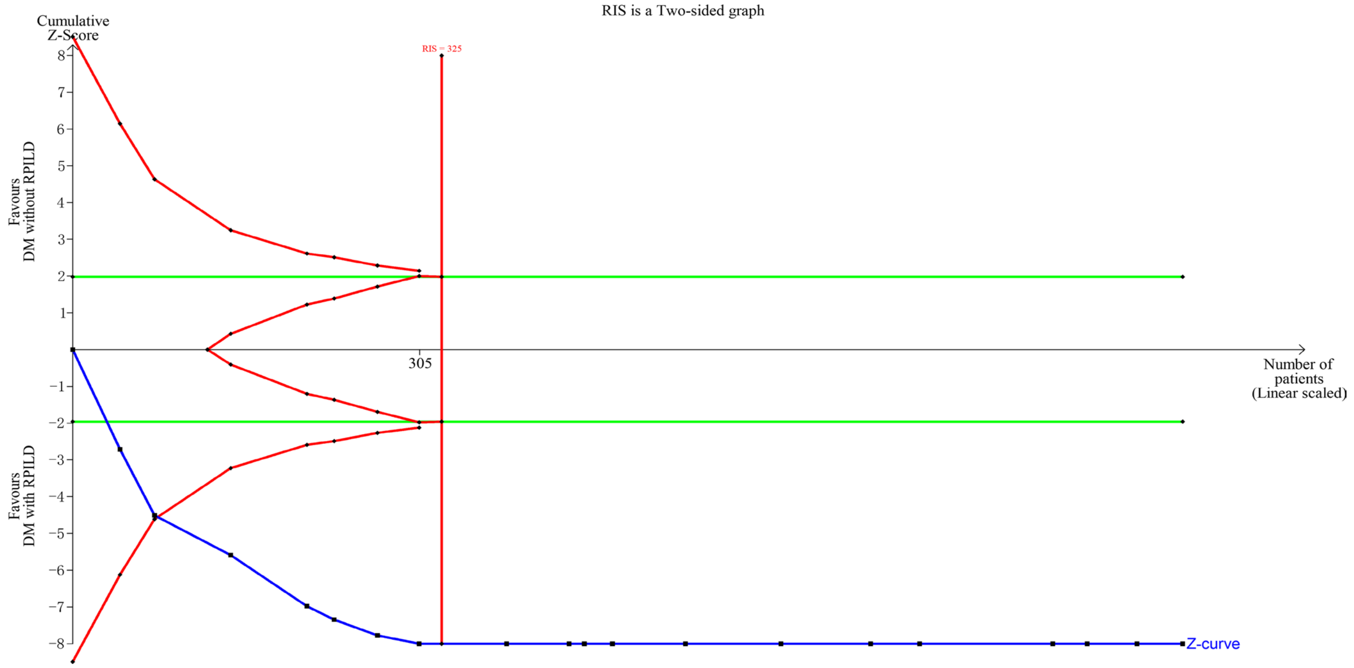

Supplementary Figure 13: Trial sequential analysis for the association between anti-MDA5 antibody and DM-RPILD. A diversity adjusted information size of 325 patients was calculated using α = 0.05 (two sided), β = 0.20 (80% of power), relative risk reduction = 65%, and an event proportion of 14% in the control arm. Complete blue line represents the cumulative Z-curve, which crossed both the conventional boundary (complete green line) and the trial sequential monitoring boundary (complete red line).

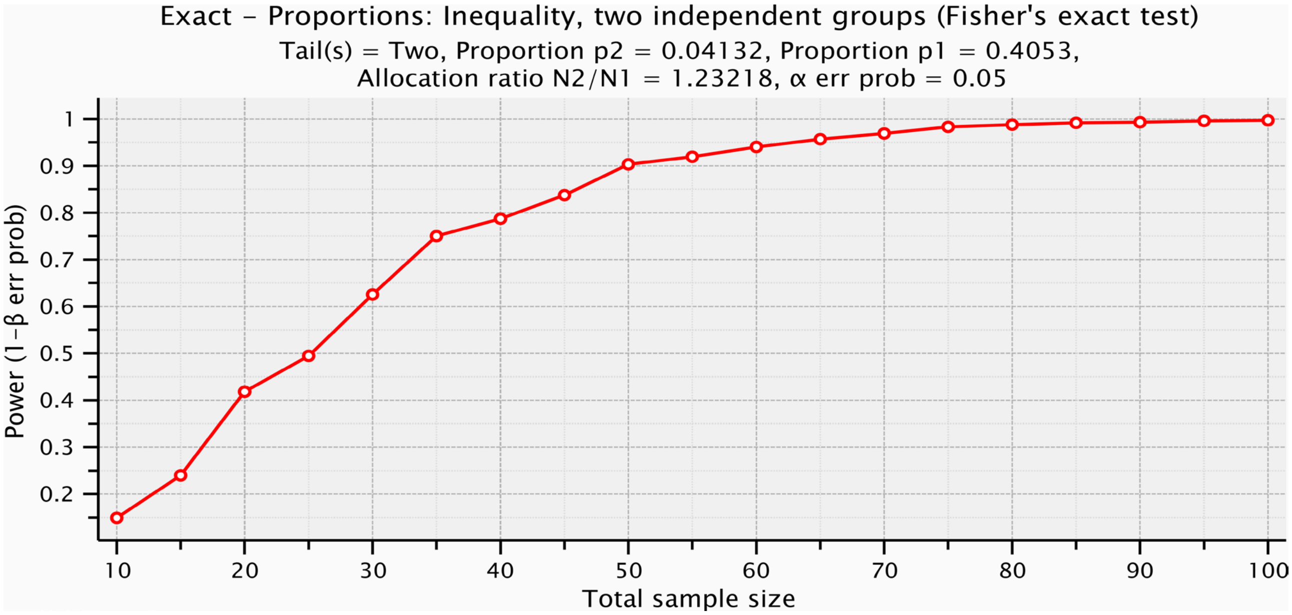

Supplementary Figure 14: The total sample size-power plot for a range of values when calculating the statistical power of the included publications with 491 DM with ILD versus 605 DM without ILD.

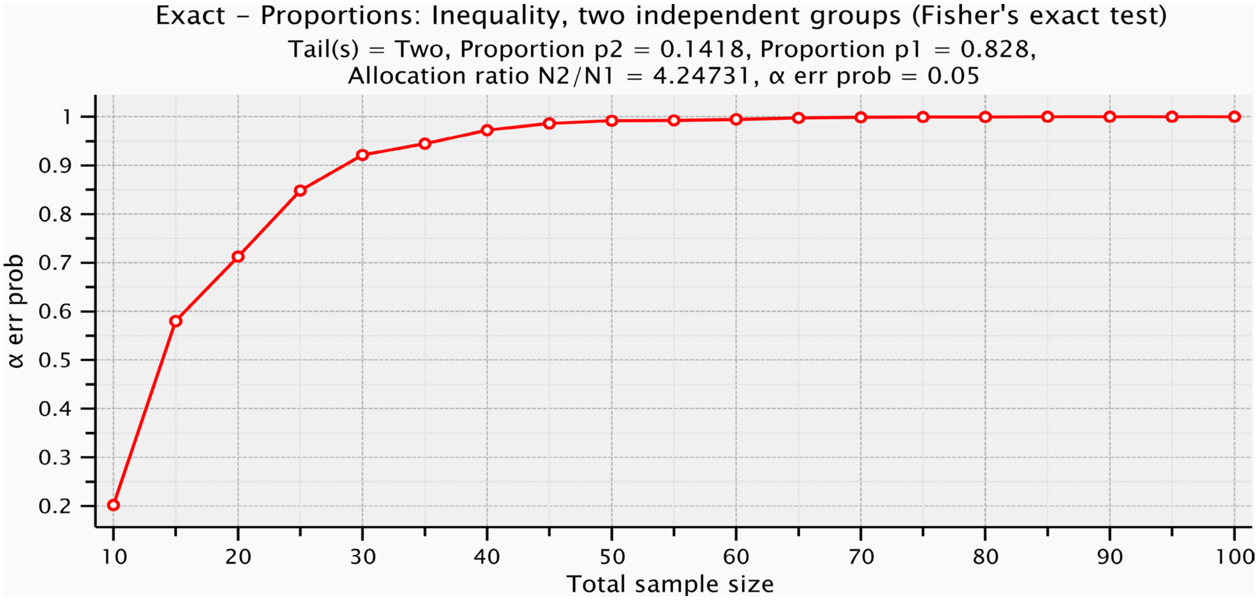

Supplementary Figure 15: The total sample size-power plot for a range of values when calculating the statistical power of the included publications with 186 DM with RPILD versus 790 DM without RPILD.

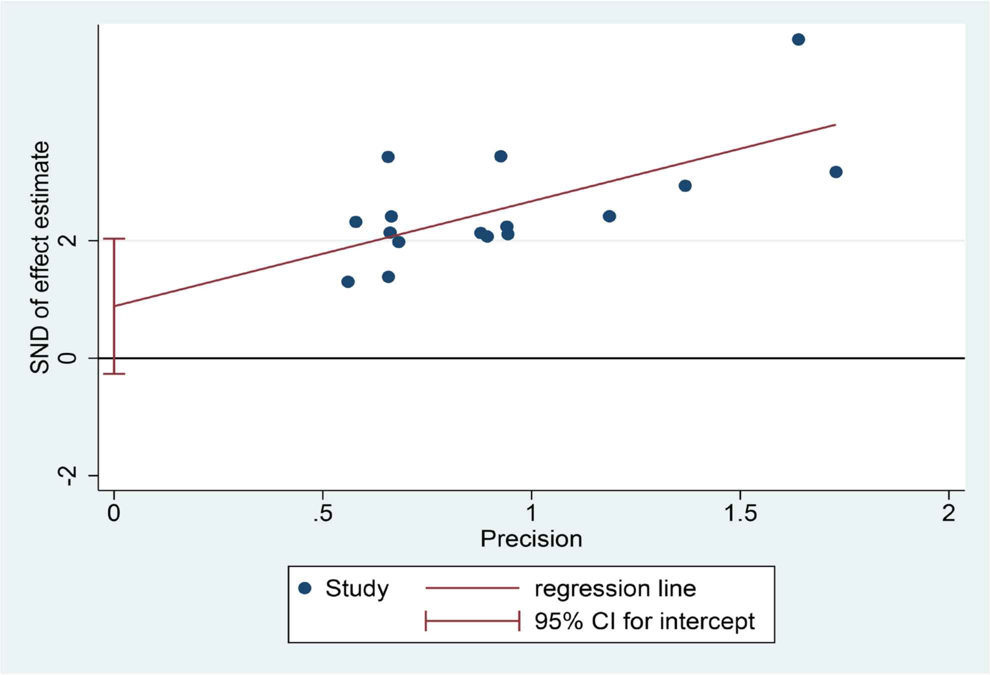

Supplementary Figure 16: Forest plot of publication bias in 491 DM with ILD versus 605 DM without ILD (*P* = 0.121).

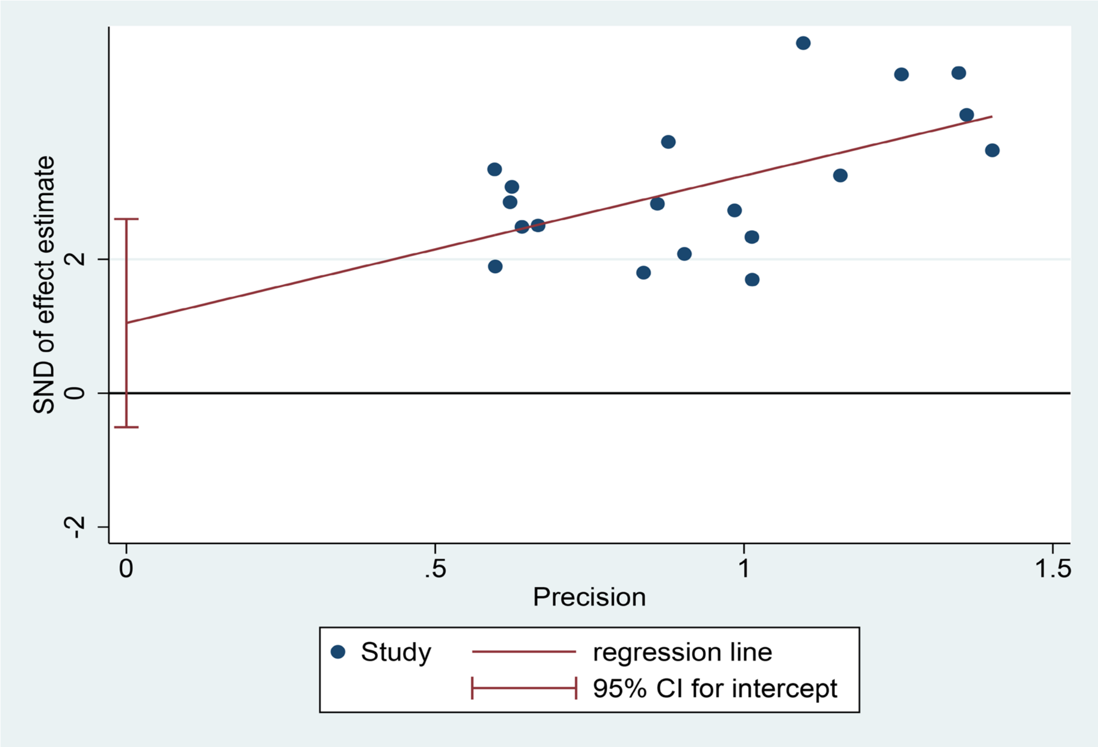

Supplementary Figure 17: Forest plot of publication bias in 186 DM with RPILD versus 790 DM without RPILD (*P* = 0.173).

Supplementary Table 1: The main characteristics of the included publications with 491 DM with ILD versus 605 DM without ILD

First author	Disease type	Cases	Ethnicity	Method	DM with ILD		DM without ILD	
					Anti-MDA5 antibody positive	Total	Anti-MDA5 antibody positive	Total
Fujikawa 2009	DM	30	Japanese	immunoprecipitation using radiolabeled K562 cell extracts	8	23	0	7
Hoshino 2010	DM	61	Japanese	immunoprecipitation using biotinylated recombinant proteins	19	32	1	29
Kang 2010	classic DM	38	Korean	immunoprecipitation using [35S]methinine-labelled HeLa cell extracts	6	12	3	26
Nakashima 2010	DM	37	Japanese	immunoprecipitation using [35S]methinine-labelled HeLa cell extracts	12	25	1	12
Fiorentino 2011	DM	77	Caucasian/Latino/ Pacific Islander/ Asian/African American	immunoprecipitation using [35S] methionine-labeled proteins	6	16	4	61
Hamaguchi 2011	DM	376	Japanese	immunoprecipitation using [35S]methinine-labelled K562 cell extracts	40	149	3	227
Ikeda 2011	DM	55	Japanese	immunoprecipitation using HeLa cells extracts and K562 cells extracts	5	20	1	35
Kobayashi 2011	JDM	13	Japanese	ELISA using recombinant MDA5 as an antigen source	5	6	0	7
Cao 2012	DM	64	Chinese	ELISA using recombinant MDA5 as an antigen source	15	46	0	18
Koga 2012	DM	79	Japanese	immunoprecipitation using extracts of leukaemia cell line K562	16	53	1	26
Chen 2013	DM	43	Chinese	ELISA using recombinant MDA5 as an antigen source	26	36	0	7
Ceribelli 2014	DM	34	European Caucasian	ELISA using recombinant MDA5 as an antigen source	3	7	2	27
Mateos 2014	CADM	11	Spanish	immunoprecipitation using K562 cell extracts	1	1	2	10
Horai 2015	DM	30	Japanese	ELISA using recombinant MDA5 as an antigen source	10	19	0	11
Kobayashi 2015	JDM	44	Japanese	immunoprecipitation using 35S-labeled HeLa cell extract	18	22	0	22
Narang 2015	DM	104	white or Asian or African American or Hispanic	immunoprecipitation using [35S] methionine-labeled proteins	9	24	7	80

DM, dermatomyositis; ILD, interstitial lung disease; JDM, juvenile dermatomyositis; ELISA, enzyme-linked immunosorbent assay; MDA5, melanoma differentiation associated gene5.

Supplementary Table 2: The main characteristics of the included publications with 186 DM with RPILD versus 790 DM without RPILD

First author	Disease type	Cases	Ethnicity	Method	DM with RPILD		DM without RPILD	
					Anti-MDA5 antibody positive	Total	Anti-MDA5 antibody positive	Total
Sato 2005	DM	42	Japanese	immunoprecipitation using radiolabeled K562 cell extracts	4	6	4	36
Fujikawa 2009	DM	30	Japanese	immunoprecipitation using radiolabeled K562 cell extracts	8	10	0	20
Sato 2009	DM	67	Japanese	immunoprecipitation using 35S-labeled HeLa cell extract	14	17	13	50
Sato 2009	DM	67	Japanese	ELISA using recombinant MDA5 as an antigen source	14	17	9	50
Gono 2010	DM-ILD	24	Japanese	immunoprecipitation using 35S-labeled HeLa cell extract	10	12	4	12
Kang 2010	classic DM	38	Korean	immunoprecipitation using [35S]methinine-labelled HeLa cell extracts	4	4	5	34
Nakashima 2010	DM	37	Japanese	immunoprecipitation using [35S]methinine-labelled HeLa cell extracts	7	8	6	29
Fiorentino 2011	DM	77	Caucasian/Latino/ Pacific Islander/ Asian/African American	immunoprecipitation using [35S] methionine-labeled proteins	2	5	8	72
Ikeda 2011	DM	55	Japanese	immunoprecipitation using HeLa cells extracts and K562 cells extracts	3	3	3	52
Kobayashi 2011	JDM	13	Japanese	ELISA using recombinant MDA5 as an antigen source	3	3	2	10
Tanizawa 2011	DM-ILD	25	Japanese	immunoprecipitation using [35S]methinine-labelled HeLa cell extracts	5	6	7	19
Cao 2012	DM	64	Chinese	ELISA using recombinant MDA5 as an antigen source	9	10	6	54
Chen 2012	DM	84	Chinese	ELISA using recombinant MDA5 as an antigen source	15	17	4	67
Koga 2012	DM	79	Japanese	immunoprecipitation using extracts of leukaemia cell line K562	12	16	5	63
Chen 2013	DM	43	Chinese	ELISA using recombinant MDA5 as an antigen source	10	11	16	32
Horrillo 2014	DM	117	Mediterranean	immunoblot using recombinant MDA5	8	11	6	106
Horai 2015	DM	30	Japanese	ELISA using recombinant MDA5 as an antigen source	10	11	0	19
Kobayashi 2015	JDM	44	Japanese	immunoprecipitation using 35S-labeled HeLa cell extract	8	8	10	36
Xu 2016	CADM	40	Chinese	ELISA using recombinant MDA5 as an antigen source	8	11	4	29

DM, dermatomyositis; RPILD, rapidly progressive interstitial lung disease; DM-ILD, dermatomyositis-associated interstitial lung disease; JDM, juvenile dermatomyositis; CADM, clinically amyopathic dermatomyositis; ELISA, enzyme-linked immunosorbent assay; MDA5, melanoma differentiation associated gene5.
